# MiPRIME: an integrated and intelligent platform for mining primer and probe sequences of microbial species

**DOI:** 10.1093/bioinformatics/btae429

**Published:** 2024-07-02

**Authors:** Zhiming Zhang, Jing Ren, Lili Ren, Lanying Zhang, Qubo Ai, Haixin Long, Yi Ren, Kun Yang, Huiying Feng, Sabrina Li, Xu Li

**Affiliations:** Research and Development Department, Coyote Bioscience (Beijing) Co., Ltd., Building 22, Zone 3, Gaolizhang Road, Haidian District, Beijing, 10095, China; Research and Development Department, Coyote Bioscience (Beijing) Co., Ltd., Building 22, Zone 3, Gaolizhang Road, Haidian District, Beijing, 10095, China; Equipment technology research institute, Science and Technology Research Center of China Customs, Tianshuiyuan street No. 6, Chaoyang District, Beijing, 100026, China; Research and Development Department, Coyote Diagnostics Lab (Beijing) Co., Ltd., Building 22, Zone 3, Gaolizhang Road, Haidian District, Beijing, 100095, China; Research and Development Department, Coyote Bioscience (Beijing) Co., Ltd., Building 22, Zone 3, Gaolizhang Road, Haidian District, Beijing, 10095, China; Research and Development Department, Coyote Diagnostics Lab (Beijing) Co., Ltd., Building 22, Zone 3, Gaolizhang Road, Haidian District, Beijing, 100095, China; Research and Development Department, Coyote Bioscience (Beijing) Co., Ltd., Building 22, Zone 3, Gaolizhang Road, Haidian District, Beijing, 10095, China; Research and Development Department, Coyote Bioscience (Beijing) Co., Ltd., Building 22, Zone 3, Gaolizhang Road, Haidian District, Beijing, 10095, China; Research and Development Department, Coyote Bioscience (Beijing) Co., Ltd., Building 22, Zone 3, Gaolizhang Road, Haidian District, Beijing, 10095, China; Research and Development Department, Coyote Bioscience (Beijing) Co., Ltd., Building 22, Zone 3, Gaolizhang Road, Haidian District, Beijing, 10095, China; Research and Development Department, Coyote Bioscience (Beijing) Co., Ltd., Building 22, Zone 3, Gaolizhang Road, Haidian District, Beijing, 10095, China

## Abstract

**Motivation:**

Accurately detecting pathogenic microorganisms requires effective primers and probe designs. Literature-derived primers are a valuable resource as they have been tested and proven effective in previous research. However, manually mining primers from published texts is time-consuming and limited in species scop.

**Results:**

To address these challenges, we have developed MiPRIME, a real-time **Mi**crobial **Pr**imer **M**ining platform for primer/probe sequences extraction of pathogenic microorganisms with three highlights: (i) comprehensive integration. Covering >40 million articles and 548 942 organisms, the platform enables high-frequency microbial gene discovery from a global perspective, facilitating user-defined primer design and advancing microbial research. (ii) Using a BioBERT-based text mining model with 98.02% accuracy, greatly reducing information processing time. (iii) Using a primer ranking score, ${PR}_{\mathrm{score}}$, for intelligent recommendation of species-specific primers. Overall, MiPRIME is a practical tool for primer mining in the pan-microbial field, saving time and cost of trial-and-error experiments.

**Availability and implementation:**

The web is available at {{https://www.ai-bt.com}}.

## 1 Introduction

Microbial detection is a crucial technology for healthy safety fields such as clinical diagnosis (Lee *et al.* 2021), food safety, environmental monitoring, and biotechnology (Li *et al.* 2023, Nachega *et al.* 2023). Accurate detection of pathogenic microorganisms requires well-designed primers and probes that can specifically and sensitively identify and amplify the target sequences. However, primer and probe design remain a challenging process/technology that involves various evaluation indices such as sequence specificity, thermodynamic stability, secondary structure, and multiplex compatibility. Several algorithm-based software tools have been developed to assist the primer and probe design, such as Primer3 (Koressaar and Remm 2007, Untergasser *et al.* 2012), Primer-BLAST (Ye *et al.* 2012), and QuantPrime (Arvidsson *et al.* 2008), etc. However, due to the complexity and diversity of microorganisms, these tools are limited by predefined databases, lack of validation information, and uncertain experimental performance.

Literature-curated primers are valuable reference materials for primer and probe design, with comprehensive information, experimental validation, and high success rate (García-Remesal *et al.* 2010). However, manually screening through thousands of references to target microbe primers is highly time-consuming and laborious. Besides, existing literature-sourced primer and probe platforms are limited to specific species or regions, such as PrimerBank (Spandidos *et al.* 2010, Wang *et al.* 2012) for human and mouse primers, ProbeBase (Loy *et al.* 2003, Loy *et al.* 2007, Greuter *et al.* 2016) for ribosomal RNA genes, LCPDb-ARG (Gorecki *et al.* 2019) for antibiotic resistance genes, and MRPrimerV (Kim *et al.* 2017) for RNA viruses. Moreover, the specificity evaluation of literature-derived primers and probes is generally insufficient among multiple output sequences of the same microbe species.

In this study, we constructed a novel real-time AI (artificial intelligence) based microbial primer mining platform, MiPRIME (https://www.ai-bt.com). It extracts species-specific primer and probe sequences from literature with three highlights: First, MiPRIME is an integrated platform that captures full-species primers from all full-text literature in real-time. To enable this, we constructed three databases as data mining references: a literature database with over 40 million abstracts or full texts, a species-wide genome database covering 548 942 species, and a database focused on antimicrobial resistance and virulence factor genes. These databases facilitated massive-scale data mining, overcame the limitation of species, and made the detection of pathogen-specific nucleic acid targets possible. Second, MiPRIME platform uses a BioBERT (Bidirectional Encoder Representations from Transformers for Biomedical Text Mining) (Lee *et al.* 2020) -based text mining model to recognize the species-specific primers accurately. This text mining model was refined with high performance on a self-built manually annotated corpus, which significantly reduces the time for manual reading and information processing. Third, comprehensive consideration multi-features of primers, a ${PR}_{\mathrm{score}}$ is used in MiPRIME to automatically recommend species-specific primers. These suggested primers have been experimentally validated with high specificity, greatly reducing the cost of trial and error. Also, gathering the microbial high-frequency genes from a global perspective, MiPRIME platform provides documentary evidence for user-defined primer design. In summary, the MiPRIME platform is currently the only species-wide primer and probe sequence mining platform in the world. The work of the MiPRIME platform is innovative and forward-thinking, filling a gap in the pan-microbial field of literature-sourced primer platforms and providing a powerful and user-friendly tool for scientific microbial detection and diagnosis.

## 2 Materials and methods

### 2.1 Data collection and database construction

#### 2.1.1 Literature collection and processing

We constructed an Elasticsearch (ES)-based literature database with >35 million abstracts collected from PubMed and 8.9 million full-text articles from PubMed Central^®^ (PMC, https://www.ncbi.nlm.nih.gov/pmc/). The python package lxml was then used to parse the full text of each paper for the title, abstract, authors, and publication date. The literature database can be freely accessed at http://106.37.92.187:1234/miprimer/.

#### 2.1.2 A species-wide database with reference genomes

A species-wide genome database was constructed to ensure species-specific primers through sequence alignment. Overcoming the limited number of species, we collected >548 942 microbial species from the Taxdb database (https://www.ncbi.nlm.nih.gov/Taxonomy/) (Schoch *et al.* 2020), including 7180 viruses, 24 784 bacteria, 55 881 fungi, etc. Reference genome sequences for all species were obtained from GeneBank (https://ftp.ncbi.nlm.nih.gov/genomes/genbank/) (Benson *et al.* 2012) or Refeq (https://ftp.ncbi.nlm.nih.gov/genomes/refseq/) (O'Leary *et al.* 2016). The selection criteria for the standard genome sequences of each species were as follows: First, reference genomes were preferred for species with multiple versions of the genome, and representative sequences were used as their reference genomes for species without reference genomes. Second, complete genome assembly was preferred, followed by chromosome-level genome assembly. Otherwise, a complete genome was randomly selected. These genome sequences were stored in an ES-based species-wide database.

#### 2.1.3 A database of antibiotic resistance and virulence genes

Since resistance and virulence of pathogenic microorganisms are important in pathogen detection (Bradley *et al.* 2019), a database of 11 532 virulence genes and 5170 antimicrobial resistant (AMR) genes was constructed for gene classification and annotation. The AMR genes and sequences were obtained from the Comprehensive Antibiotic Resistance Database (CARD, https://card.mcmaster.ca/) (Alcock *et al.* 2019, Alcock *et al.* 2023), whereas the virulence genes and sequences were received from the Virulence Factor Database (VFDB, http://www.mgc.ac.cn/VFs/main.htm) (Chen L *et al.* 2005). All the data were saved in a MySQL database.

#### 2.1.4 A Corpus of artificially labeled microbial species and primer sequences

We construct a self-built Microbial Primer Corpus (MPC) with expert annotations for the task of text mining model optimization for microbiological primers. From 2016 to 2022, 500 full-text papers related to “microorganisms” AND “primers” were randomly screened in the PMC. Following the corpus tagging rules (detailed in Supplementary Information S1), each article was manually annotated with forward primers, reverse primers, probes, species, genes, virulence genes, resistance genes, etc. In total, a corpus with 8458 different types of labels was constructed as the standard for the following Natural Language Processing (NLP) models.

### 2.2 A text mining model for the primer and probe sequence

#### 2.2.1 Data cleaning

To identify sequences of primers and probes, we used two python packages, lxml and pubmed_parser, to parse an XML file of a paper and obtain table information. Specially, for primers target microbial antimicrobial resistance and virulence gene, we selected the papers with keywords such as “Drug Resistance, Microbial” [Mesh], or “Virulence” [Mesh] first.

#### 2.2.2 Sequences of primers and probes extracted from literature

Primer and probe sequences were extracted from the full text and tables using a regular expression pipeline that matches strings of length 8–30 bp. These strings are composed of four nucleotide bases: adenine (A), cytosine (C), guanine (G), and thymine (T), as well as wildcard characters (such as B, D, H, K, M, N, S, V, W, and Y).

#### 2.2.3 The identification of species and genes

Microbial species and genes were identified and extracted using a hybrid approach combining dictionary-based, BioBERT-based and GnormPlus (Wei *et al.* 2015) methods. The latter is a common integrative NER (Named Entity Recognition) approach for tagging genes, gene families, and protein domains.

#### 2.2.4 Primer targeting relation extraction and correction

The relations between primers and species were extracted by semantic similarity based on word co-occurrence analysis. Considering the uncertainty of these relationships, we used a sequence alignment tool, BLASTn (Johnson *et al.* 2008), to verify the targeted segment, and a qcovhsp (query coverage per high scoring pair) to screen the confidence species-primer connections. Details of the screening criteria were given in Supplementary data S2. Similar to what was described above, interactions between primers and resistance or virulence genes were identified and corrected.

### 2.3 The accuracy of text mining models

The MPC corpus with 500 full texts was divided into three datasets, 300, 100, and 100 articles as train, development, and test sets, respectively. The train and development sets were used for parameter optimization and model building, while the test sets were used to evaluate the models. Four measurements, including accuracy, precision, recall and F1 score, were calculated to assess model performance. And the accuracies of text mining models were above 0.9. For more details, please refer to Supplementary data S3.

### 2.4 *PR*_score_: a novel evaluation index for microbial primer recommendation

Numerous variables, including the primer’s specificity, coverage, melting temperature (Tm), GC content, hairpin structure, self-dimer, cross-dimer, etc., have effects on the performance of a primer for the detection of pathogens. Taking these factors into account, we proposed a weighted primer evaluation metric, Primer Ranking score (${PR}_{\mathrm{score}}$), to thoroughly assess the performance of a primer. Literature-sourced primers on MiPRIME were ranked in descending order by ${PR}_{\mathrm{score}}$, and those with high scores were recommended for experiments. The ${PR}_{\mathrm{score}}$ was calculated using the following formula:

(1) $${PR}_{\mathrm{score}}=P_{\mathrm{specificity}}+P_{\mathrm{coverage}}+L_{\mathrm{Tm}}+L_{\mathrm{GC}\%}-N_{\mathrm{hairpin}}-N_{\mathrm{dimer}}+N_{\mathrm{literature}}\times {\left(\frac{P_{\mathrm{specificity}}}{100}\right)}^{2}$$

where “$P_{\mathrm{specificity}}$” refers to the percentage of the primers successfully mapped to the reference genome; “$P_{\mathrm{coverage}}$” refers to the percentage of the sequences successfully aligned; “$L_{\mathrm{Tm}}$” refers to whether the value of Tm is between 50°C and 80°C (Rozen and Skaletsky 2000), where 1 is yes, 0 is no; “$L_{\mathrm{GC}\%}$” refers to whether the GC content is between 40–60% (Lohoff *et al.* 2021, Takei *et al.* 2021), also 1 is yes, 0 is no; “$N_{\mathrm{hairpin}}$” refers to the sum of forward primer hairpin score level and reverse primer hairpin score level; the primer hairpin score level refers to whether the Δ*G* of hairpin calculated by MFEprimer (Wang *et al.* 2019) is <4, when 1 is yes, 0 is no. “$N_{\mathrm{dimer}}$” refers to the sum of forward primer self-dimer score level, reverse primer self-dimer score level, and cross-dimer score level of forward/reverse primer score; the dimer score level refers to whether the Δ*G* of hairpin calculated by MFEprimer is <4, when 1 is yes, 0 is no. “$N_{\mathrm{literature}}$” refers to the number of supported articles. Here we modified the literature weights by the specificity of the primers, since primers with lower specificity usually have higher numbers of supported references.

### 2.5 Development tools

The MiPRIME web server was designed in a user-friendly manner which can be accessible via computers, tablets, and mobile phones at https://www.ai-bt.com. The front end of MiPRIME platform was developed using CSS, PHP, JavaScript, HTML, XML, etc., whereas the backend is comprehended using MySQL, ES, Python, etc.

## 3 Results

### 3.1 MiPRIME: an integrated primer text mining platform for all species

**Figure 1.**
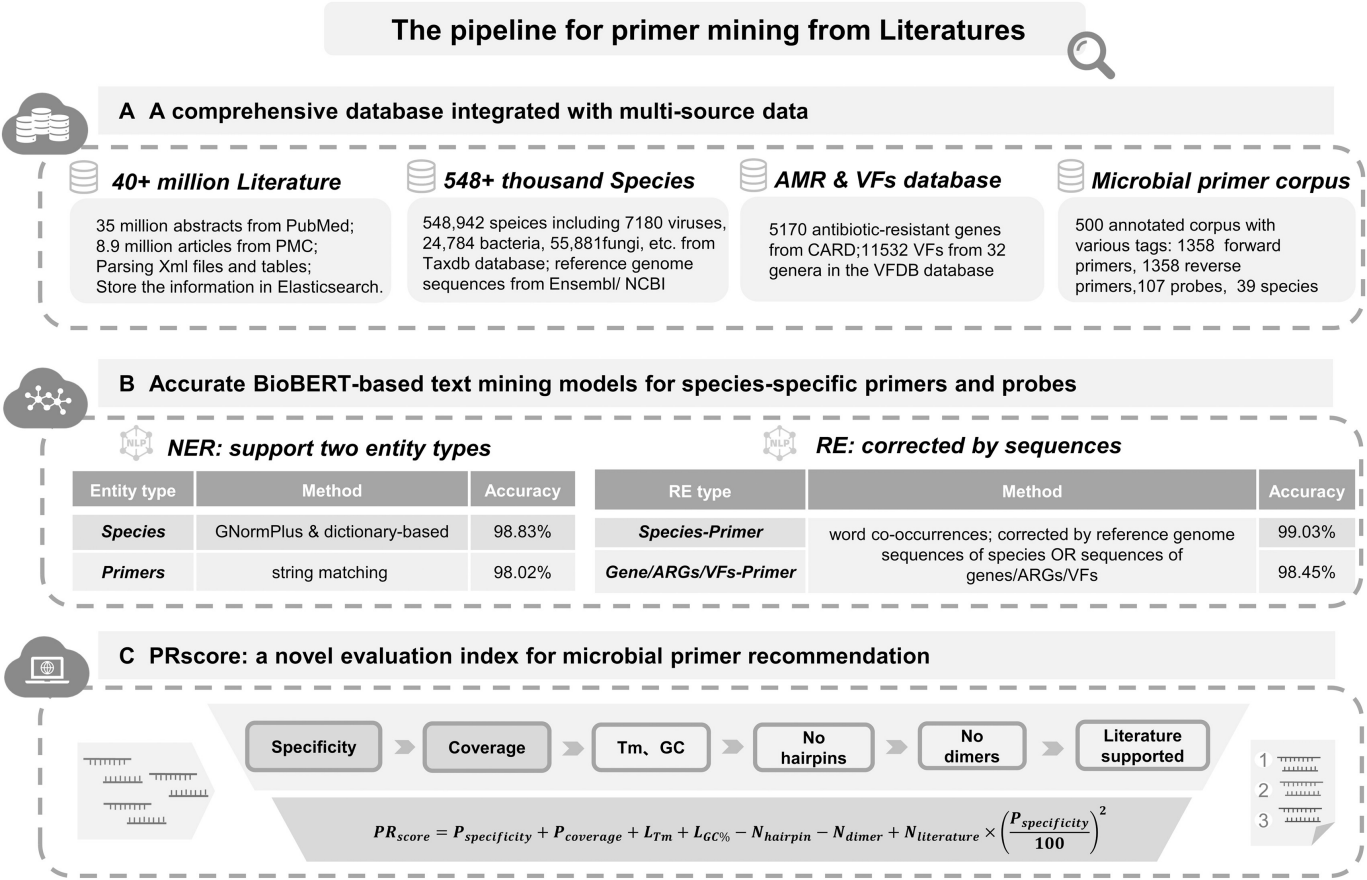
The pipeline of MiPRIME platform. The pipeline of MiPRIME platform consists of three major steps: (A) A comprehensive in-house database as a data source for worldwide primer/probe information including: (i) a biomedical literature database with 40 million open-access articles; (ii) a pan-species sequence database with 548 942 microorganism reference genomes to validate the accuracy of discovered primer/probe sequences; (iii) AMR and VFs database for the annotation the resistance and toxin genes of target microbial species; and (iv) a microbial primer corpus for the optimization of BioBERT-based model. (B) Accurate BioBERT-based text mining models for species-specific primers and probes. (C) ${PR}_{\mathrm{score}}$: a novel index for microbial primer recommendation

In this study, we construct a rare literature-derived primer/probe mining platform, MiPRIME, that enables users to obtain high-quality primers and probe sequences for any target microbial species from 40 million published literature texts. As shown in Fig. 1, the construction of the MiPRIME platform consists of three major steps. First, database construction, to construct a Pan-species primer/probe mining platform we built a comprehensive in-house database as a data source for worldwide primer/probe information (Fig. 1A), which including 3 databases of (i) a biomedical literature database with 40 million open-access articles that provide sources of worldwide published pan-species microbial primer/probe sequences; (ii) a pan-species sequence database with 548 942 microorganism reference genomes to validate the accuracy of the discovered primer/probe sequences; and (iii) AMR and virulence factors (VFs) database for the annotation the resistance and toxin genes of target microbial species. Second, primer/probe extraction model (Fig. 1B), to accurately identify targeting primer/probe information from massive literatures, we developed a BioBERT-based text mining model that was fine-tuned using our in-house manually annotated corpus. In this model, entity recognition methods such as regular expression matching, dictionary-based matching, and GNormPlus (Wei *et al.* 2015) were used for the identification of primers and species. And the associations between them were supported and confirmed by both word co-occurrence analysis and sequence alignment. Third, best primer/probe recommendation (Fig. 1C), for auto-recommendation of species-specific primers with best coverage and high PCR success rates, we proposed a primer evaluation metric, ${PR}_{\mathrm{score}}$, which is a weighted sum of primer specificity, coverage, GC content, Tm, hairpin, dimer, etc. Usually, primers with the highest ${PR}_{\mathrm{score}}$ were preferred.

### 3.2 The main functions of MiPRIME platform: a case of *Streptococcus pyogenes*

**Figure 2.**
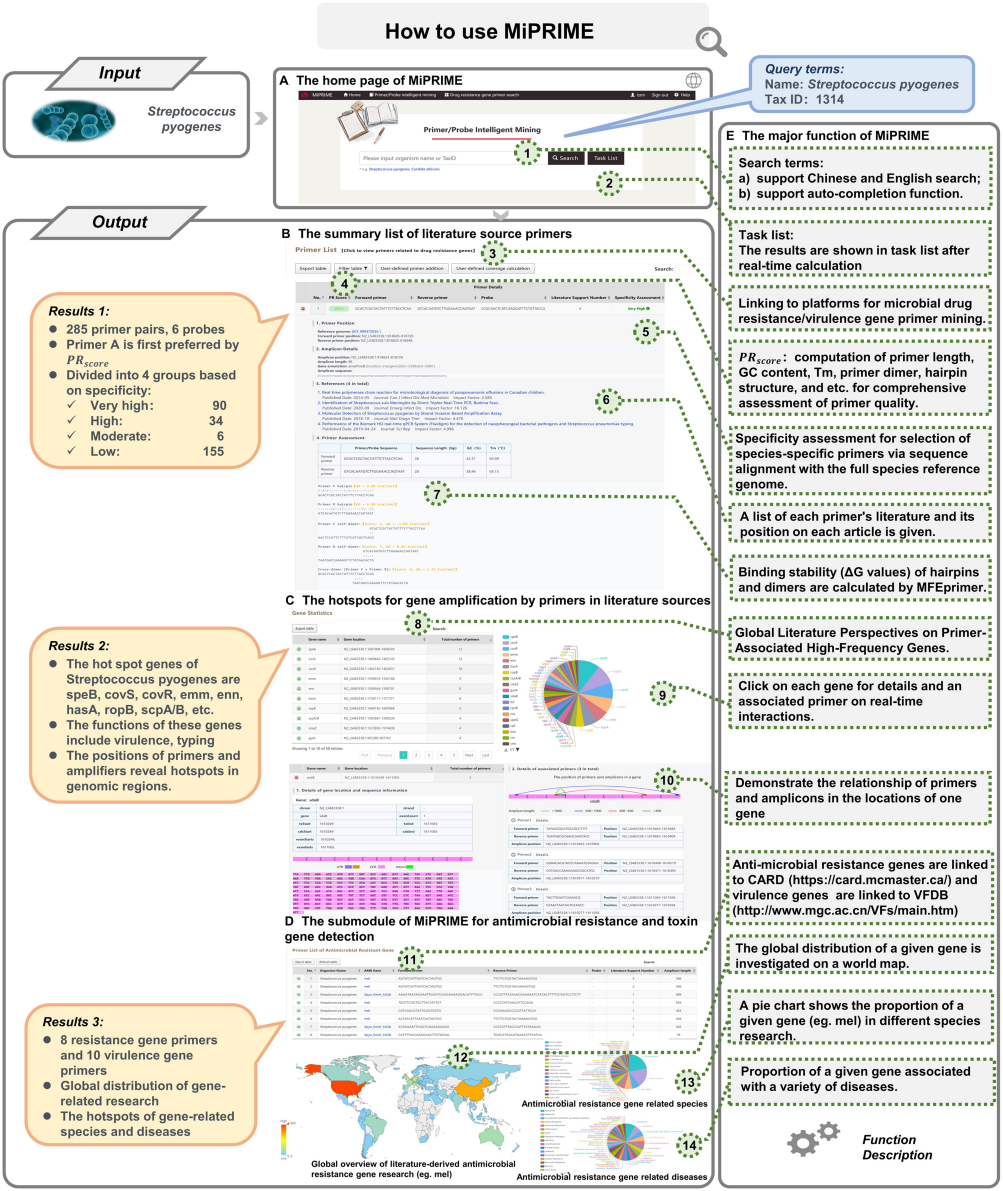
The usage and functionality of the MiPRIME platform. (A) home page; (B) the summary list of literature-sourced primers and probes, the primers here were sorted by PRscore and divided into four group by specificity level; (C) the detection hotspots of a given species from the perspective of global literature; (D) the submodule of MiPRIME for drug resistance and toxin gene detection; (E) the major function of MiPRIME

Here we provide a case study on the usage and functionality of the MiPRIME platform, using *Streptococcus pyogenes* [also known as *group A streptococci* (*GAS*)] as an example (Fig. 2). The MiPRIME platform allows users to enter a given species name or Tax ID for text mining primers (Fig. 2A). The search box features an autocomplete function for convenience. After a real-time calculation, users can review the task results in their personal center. The results here were grouped into three panels: The first panel (upper panel) presents a comprehensive list of literature-derived primers ranked by ${PR}_{\mathrm{score}}$ (Fig. 2B). For *GAS*, 285 primer pairs and 6 probes were collected from 3685 articles. According to ${PR}_{\mathrm{score}}$, primer (Forward primer: GCACTCGCTACTATTTCTTACCTCAA; Reverse primer: GTCACAATGTCTTGGAAACCAGTAAT) and probe (CCGCAACTCATCAAGGATTTCTGTTACCA) were the preferred option. While for species-specific detection, additional 90 primers with high levels of specificity were proposed as optional primers. The second panel (lower panel) reveals the detection hotspots of a given species from the perspective of global literature (Fig. 2C). These hot-spot genes of *GAS* include speB (Deng *et al.* 2022), covS, covR, emm, enn, hasA, ropB, scpA/B, etc. Some of these genes are virulence genes that are highly correlated with microbial pathogenicity, while others are commonly used for strain typing. The statistics of these hotspot primer/probe design regions will assist researchers in designing their own primers/probes. The third panel is a submodule of MiPRIME for mining the primer/probe sequences of resistance and toxin genes. Here, a total of 3167 primers for 368 resistance genes were identified and extracted from all published literature, revealing the general rules of microbial resistance, pathogenicity, and epidemics (see Supplementary data S4 and S5). In the case of *GAS*, eight resistance gene primers were observed from 2001 articles (Fig. 2D). Among these genes, gene mel had the largest amount of supporting literature. We linked this antibiotic resistance gene to an independently developed AI literature platform (http://106.37.92.187:1234/miprimer/) to directly observe its global distribution, associated species, and diseases. This highlights the significance of detecting this gene.

Although we use GAS as an example in this study, the MiPRIME platform has widely applicability across various microorganisms including bacteria, fungi, and viruses. To validate MiPRIME's performance and reliability, we selected three representative species: *severe acute respiratory syndrome coronavirus 2* (*Covid-19*), *Candida albicans*, and *Chlamydia trachomatis*, to validate both high-specific and low-specific primers by RT-qPCR (Real-time quantitative polymerase chain reaction). Furthermore, we assessed primer specificity using *human coronavirus 229 E* (*HCoV-229E*), *Candida tropicalis*, and *Chlamydia pneumoniae*. Our analysis of amplification curves, melting curves, and agarose gel electrophoresis revealed that primers with high ${PR}_{\mathrm{score}}$ are more effective in producing target-specific PCR products. Conversely, primers with low ${PR}_{\mathrm{score}}$ were associated with anomalies such as amplification of nontarget species sequences, formation of primer dimers, or absence of amplification products. Detailed experimental procedures and results can be found in Supplement data S6.

Overall, the MiPRIME platform serves as a valuable resource for researchers in the field of microbial detection and diagnosis. Its innovative approach to literature-derived primer mining, combined with its user-friendly interface and comprehensive functionality, makes it a valuable tool for advancing scientific understanding in various domains related to microbial detection.

## 4 Discussion

In this study, we developed a rare platform, MiPRIME, for real-time mining of primers and probes across all microbial species. It addresses the existing challenges in primer design by providing a comprehensive solution that combines literature mining, species-specific database integration, and advanced text mining techniques.

Our platform innovatively solves the problem of time-consuming and labor-intensive manual screening of thousands of references for targeting primers/probes. Additionally, it overcomes the limitations of existing primer platforms, which are often limited to specific species or regions, and lack validation information. By integrating multiple databases and using a refined biomedical NLP model, the MiPRIME platform enables efficient extraction of species-specific primers with high accuracy. It also offers users the flexibility to design their own primers while considering various primer features. Importantly, MiPRIME fills a significant gap in the field of literature-derived primer platforms by providing a species-wide primer mining solution. It addresses the need for comprehensive information, experimental validation, and high success rates in primer design. Furthermore, the platform statistics the primer design hotspots from a global perspective, assisting researchers in designing their own primers and gaining insights into pathogen detection.

**Figure 3.**
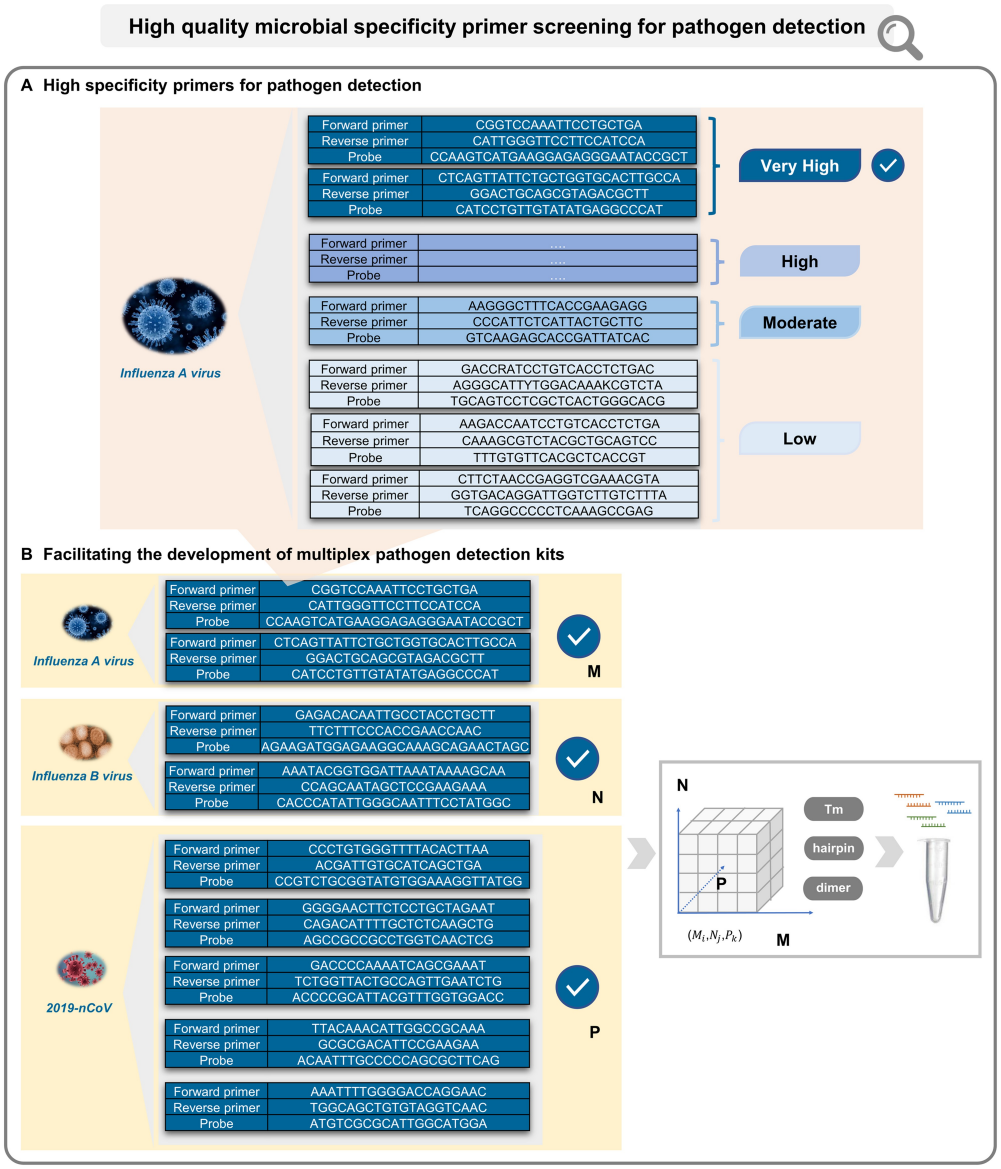
The application of the MiPRIME platform in pathogen detection. (A) For a given pathogen, recommended the high-specific primer according to ${PR}_{\mathrm{score}}\;\mathrm{or}$$P_{\mathrm{specificity}}$. (B) The potential application of multi-species pathogen detection

More interesting, these primers and genes further extend the potentially broad applications of MiPRIME, such as a given pathogen detection (Fig. 3A), or multi-species pathogen detection (Fig. 3B). In the case of the design of a multiplex respiratory pathogen panel for influenza A, influenza B and Covid-19, the MiPRIME platform allows us to collect experimentally validated species-specific primers for each species, which can be used to form a combined list for multiplex PCR primer selection; the stability of multiple primers will then be assessed through the multiple dimensions of these primers, such as Tm, hairpin structure, homodimer and heterodimer; Finally, an optimal combination of primers is recommended for multiple pathogen detection.

Overall, our platform represents a creative and efficient tool that improves primer/probe design and contributes to advancements in scientific microbial detection and diagnosis. Although the sequences of these primers/probes originate from the experimental segment of the researchers' work, rendering them reliable and valuable for beginners, it remains crucial for users to adopt a critical approach when evaluating the outcomes derived from these primers/probes, in views of some limitations: (i) due to copyright issues with certain journals, we could only include a limited number of articles with full texts. This leads to a scarcity of information regarding the sequences of primers/probes. In addition, we failed to take into account the potential bias introduced by outdated information or writing errors in the literature, necessitating caution on the part of the user when using these primer and probe sequences. (ii) the platform currently does not distinguish between different forms of PCR, such as conventional PCR, real-time PCR, nested PCR, reverse-transcription PCR, multiplex PCR, etc., therefore, users need to click the links to view the full-texts for specific details. In the future, we aim to overcome these shortcomings and enhance the user experience by improving the platform.

## Supplementary Material

btae429_Supplementary_Data

## Data Availability

The data underlying this article are available in the article and in its online supplementary material. Additionally, related data and materials can be accessed through the platform provided at https://www.ai-bt.com.
