# Supplementary material for: MiPRIME: an integrated and intelligent platform for mining primer and probe sequences of microbial species
Source: Bioinformatics. 2024 Jul 2;40(7):btae429. doi: 10.1093/bioinformatics/btae429 (PMC11246166; doi:10.1093/bioinformatics/btae429)
Supplement: btae429_Supplementary_Data [file btae429_supplementary_data.zip › SI.docx]

Supporting Information

S1 The corpus tagging rules

To ensure the accuracy and quality of the label dataset for the BioBERT-based texting model development, we constructed a Microbial Primer Corpus (MPC) with the tagging rules as follows (see details in Table S1):

**Table S1 Description of entity classes in the manually validated dataset**

| **Entity Class** | **Definition** | **Example** |
| --- | --- | --- |
| Primer | A short single-stranded DNA sequence (usually 15-30 nucleotides) which bind to a specific region of the DNA sequence for DNA amplification. | forward primer, 5′-GACCRATCCTGTCACCTCTGAC-3′  reverse primer, 5′-AGGGCATTYTGGACAAAKCGTCTA-3′ |
| Forward primers | A primer binds to the end of the sense strand of DNA and amplify the sense strand of DNA | F (5’-AGA GTT TGA TCC TGG CTC AG-3’);  forward primer, 5′-GACCRATCCTGTCACCTCTGAC-3′ |
| Reverse primers | A primer binds to the end of the antisense strand of DNA and amplify the antisense strand of DNA | R (5’-GGT TAC CTT GTT ACG ACT T-3’)  reverse primer, 5′-AGGGCATTYTGGACAAAKCGTCTA-3′ |
| Probe | A probe is a short stretch of DNA or RNA, which is designed to bind to a specific target sequence for the identification of the presence of a particular gene, RNA, or microbial organism in a sample. | probe, 5′-FAM-TGCAGTCCTCGCTCACTGGGCACGBHQ1-3′ |
| Species | The names of species are labeled by Taxdb database | Influenza A Virus |
| Gene | Genes are made up of DNA. | matrix protein 1 (M1) |
| Virulence genes | Virulence factors refer to the properties (i.e., gene products) that enable a microorganism to establish itself on or within a host of a particular species and enhance its potential to cause disease. |  |
| Resistance genes | Antibiotic resistance genes (ARGs) are genes that encode for resistance to antibiotics and other antimicrobial agents. | BLA, β-lactam resistance genes; QLN, quinolone resistance genes; FOS, fosfomycin resistance genes |

S2 The details of screening criteria of sequence alignment

The primers sequences were compared with targeting genes sequences by blastn. We selected the excellent sequence alignment results as follows: (i)the qcovhsp (query coverage per hsp) of both forward/reverse primer and their target gene was ≥ 95%;(ii) the qcovhsp of forward/reverse primer was ≥ 99.8% and target gene ≥ 80%; or the qcovhsp of forward/reverse primer was ≥ 80% and target gene ≥ 99.8%; (iii) the qcovhsp of both forward/reverse primer and target gene were ≥ 90%.

S3 The performance of text mining model on MiPRIME

The goal of the MiPRIME text mining model is to mine primers and probe sequences for a given species from numerous full texts. To create the model with high performance, MPC were randomly divided into a training set (N=300 full texts), a development set (N=100 full texts), and a test set (N=100 full texts). The number of entities such as primer (forward/reverse primer) and probes, and relations were shown in Table S2. The precision, recall and F1 of the sub models are presented in Table S3 and S4.

**Table S2** **Number of labels in MPC for NER and RE**

| **Labels** | **Counts (% of Labels on Tokens)** | | |
| --- | --- | --- | --- |
|  | **Train set** | **Validation set** | **Test set** |
| Primer | 904 | 218 | 236 |
| forward primers | 452 | 109 | 118 |
| reverse primers | 452 | 109 | 118 |
| Probe | 75 | 23 | 19 |
| Species | 28 | 9 | 11 |
| Gene | 364 | 62 | 78 |
| virulence genes | 46 | 13 | 19 |
| resistance genes | 52 | 17 | 14 |
| forward primers -reverse primers | 452 | 109 | 118 |
| Primer-species | 904 | 218 | 236 |
| Primer-genes | 904 | 218 | 236 |

**Table S3 The performance of BioBERT-based model on the Validation Set**

| **Class** | **Precision** | **Recall** | **F1** |
| --- | --- | --- | --- |
| Primer | 0.973 | 0.851 | 0.829 |
| forward primers | 0.973 | 0.851 | 0.829 |
| reverse primers | 0.973 | 0.851 | 0.829 |
| Probe | 0.99 | 0.944 | 0.943 |
| Species | 0.977 | 0.789 | 0.786 |
| Gene | 0.981 | 0.811 | 0.837 |
| virulence genes | 0.965 | 0.952 | 0.936 |
| resistance genes | 0.979 | 0.931 | 0.969 |
| forward primers -reverse primers | 0.99 | 0.932 | 0.928 |
| Primer-species | 0.993 | 0.827 | 0.815 |
| Primer-genes | 0.969 | 0.754 | 0.879 |

**Table S4 The performance of BioBERT-based model on the Test Set**

| **Class** | **Precision** | **Recall** | **F1** |
| --- | --- | --- | --- |
| Primer | 0.964 | 0.889 | 0.862 |
| forward primers | 0.964 | 0.889 | 0.862 |
| reverse primers | 0.964 | 0.889 | 0.862 |
| Probe | 0.992 | 0.958 | 0.926 |
| Species | 0.981 | 0.782 | 0.811 |
| Gene | 0.986 | 0.941 | 0.913 |
| virulence genes | 0.962 | 0.986 | 0.836 |
| resistance genes | 0.971 | 0.927 | 0.874 |
| forward primers -reverse primers | 0.984 | 0.915 | 0.899 |
| Primer-species | 0.988 | 0.939 | 0.917 |
| Primer-genes | 0.964 | 0.943 | 0.953 |

S4 The upward trend of specific primers from a global perspective

Specific primers are pivotal for PCR detection of pathogenic microorganisms. The most common cause of false positive PCR test results is insufficient specificity of the primers. The false negative results are mainly caused by the fact that while the primers enable amplification of a specific single target sequence belonging to some target species, not all. To solve the issues above, the MiPRIME platform first established a species-wide genome database covering 58,492 microorganisms; and then a sequence alignment software, blastn, was used to align the primer sequence to the species-wide reference genome for the elimination of specificity bias; finally, the primers were ranked in descending order by PRscores and the highest one was usually recommended. Also, according to $P_{specificity}$, a percentage of the primers successfully mapped to the reference which reflects the specificity levels of primers (see Method 2.3), primers with very high levels of specificity were recommended for more primers for specific detections.

Now, MiPRIME platform has completed real-time calculations for 263 species. From 102,291 articles, we obtained 11,861 primers, including 4559 high quality specific primers. These specific primers are mainly derived from the studies of different species, such as severe acute respiratory syndrome coronavirus 2 and Mycobacterium tuberculosis, which are hotspots for differential diagnosis of pathogenic microorganisms. In a global perspective, we found that the number of publications related to these specific primers showed an increasing tendency over time（p-value < 0.01, r=0.906, see Figure S1）. Among the available literature-derived primers, the COVID-19 primer (Forward Primer: GACCCCAAAATCAGCGAAAT; Reverse primer: TCTGGTTACTGCCAGTTGAATCTG; Probe: ACCCCGCATTACGTTTGGTGGACC) has the highest number of publications among specific primers, with a Staphylococcus aureus primer (Forward Primer: GACCCCAAAATCAGCGAAAT; Reverse primer: TCTGGTTACTGCCAGTTGAATCTG) in second place.


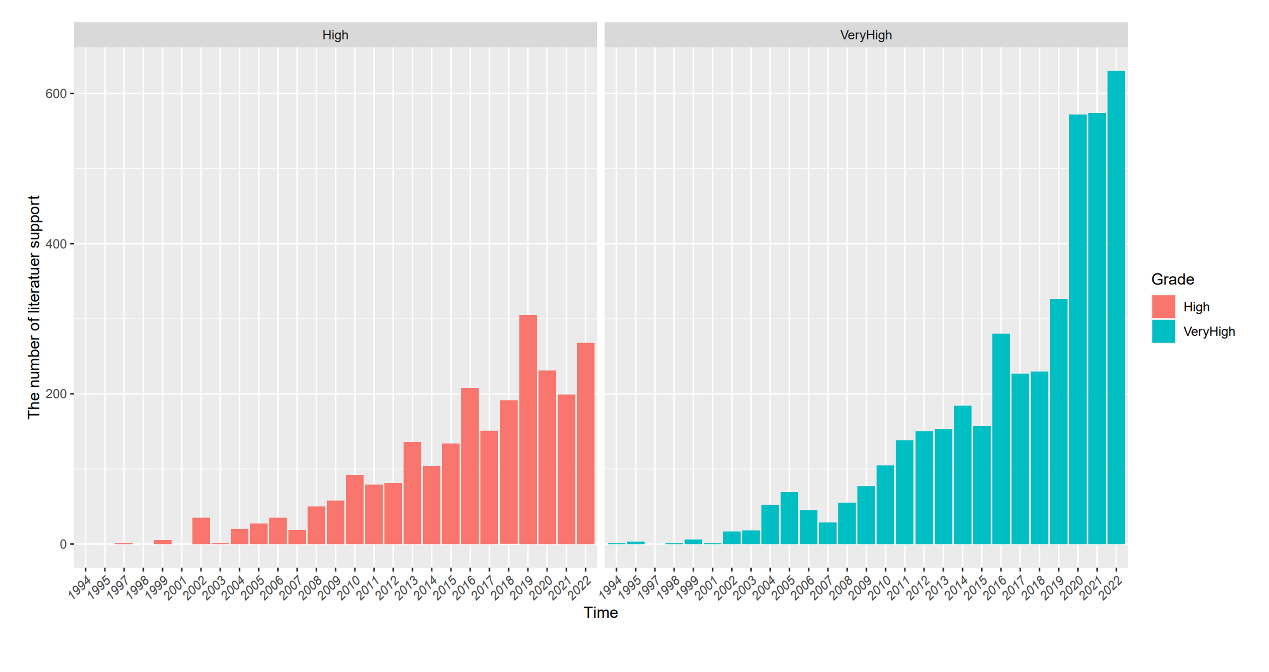


Figure S1 A worldwide rise in the number of specific primers

S5 The sub-modules for antimicrobial resistance and virulence factor genes

Microbial antibiotic resistance/ virulence genes are of interest for the detection of pathogenic microbes. To motivate the design of a primer-probe for antimicrobial resistance or virulence genes, we obtained 3167 primers for 368 resistance genes from 6074 publications and 2743 primers for 294 virulence genes from 3831 publications, revealing global patterns of microbial resistance, pathogenicity, and epidemics from a literature perspective. As shown in Figure S2, most scholars focused on a tetracycline resistance gene Hpyl_16S_TET in *Helicobacter pylori 26695*, followed by Msme_16rrsB_HGM gene in *Mycolicibacterium smegmatis*, and Cacn_16S_TET in *Cutibacterium acnes*, etc. From the perspective of article publications, primer 1 (Primer F: AGAGTTTGATCCTGGCTCAG; Primer R: GGTTACCTTGTTACGACTT) has been widely used in the amplification of Hpyl_16S_TET, supported by 274 articles. And primer 2 (Primer F: AGAGTTTGATCMTGGCTCAG; Primer R: TACGGYTACCTTGTTACGACTT) came in second with 103 papers.


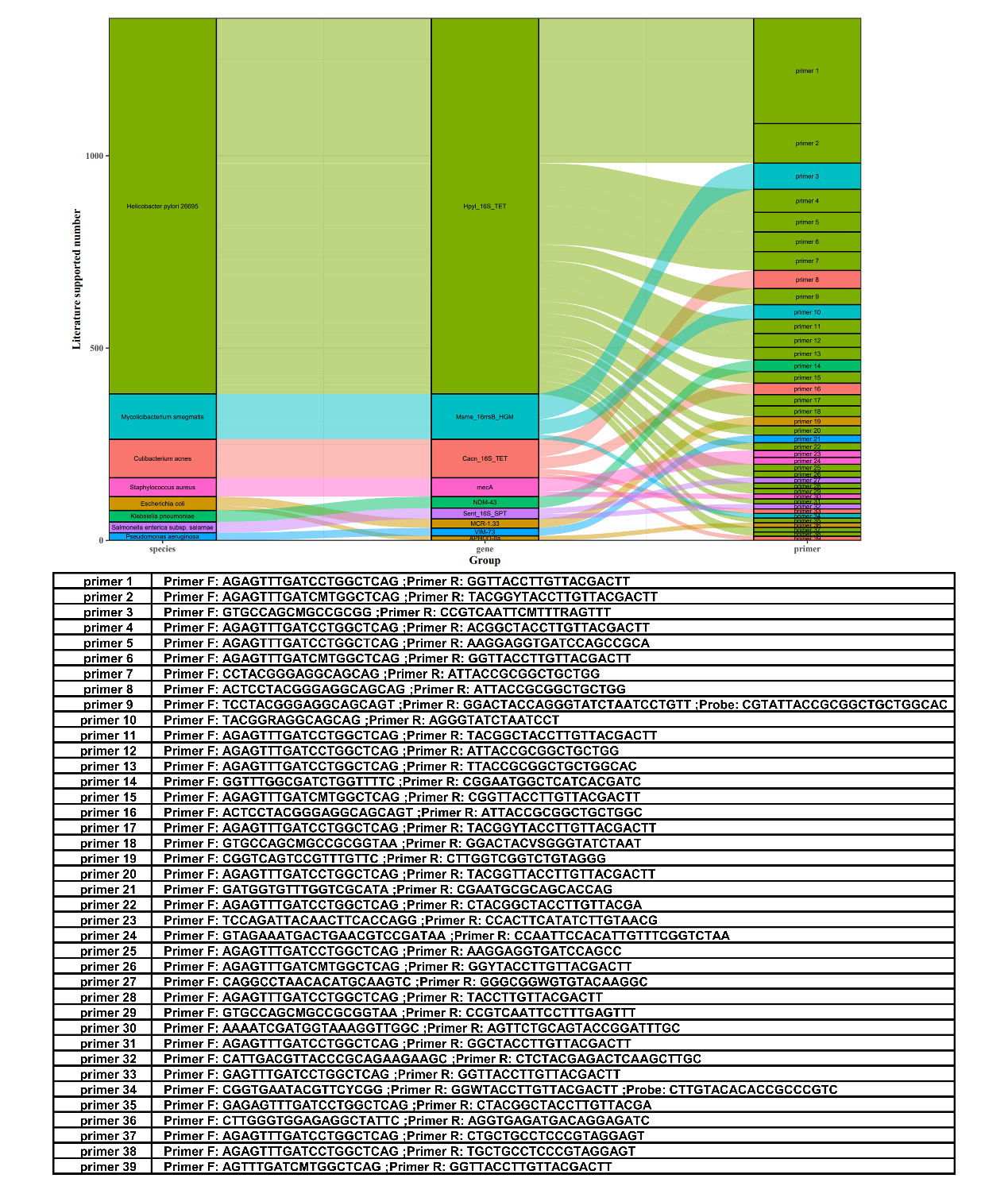


**Figure S2** **The commonly used primers for antimicrobial resistance genes**

S6 MiPRIME's performance and reliability validation by RT-qPCR

To validate the reliability of the MiPRIME platform, we used three representative species, *severe acute respiratory syndrome coronavirus 2* (*Covid-19*) (virus), *Candida albicans* (fungi), and *Chlamydia trachomatis* (bacteria), highly specific primers and non-specific primers, respectively, for RT-qPCR (Real-time quantitative polymerase chain reaction) validation. Meanwhile, *human coronavirus 229 E* (*HCoV-229*), *Candida tropicalis* and *Chlamydia pneumoniae* (*CP*) were selected to evaluate primer specificity. Amplification curve, melting curves and agarose gel electrophoresis of PCR products were used to verify the performance of PCR amplification. The fungi, bacteria, and virus used in the testing were provided by Coyote Diagnostics Lab (Beijing) Co., Ltd, with concentrations of approximately 10,000 copies/ml. All primers for RT-qPCR (see Table S5) were synthesized by Sangon Biotech (Shanghai). For additional experimental details and tables listing instruments, consumables and reagents used in this study, please see Table S6.

**Table S5 Primer sequences used for experimental verification**

| **Species** | **Primer No.** | **Primer Sequence** | **PR Score** | **Specificity** | **Amplicon** | **Gene** | **Tm** |
| --- | --- | --- | --- | --- | --- | --- | --- |
| *Covid -19* | 1 | F: GCGTTCTTCGGAATGTCG  R: TTGGATCTTTGTCATCCAATTTG | 186 | High | 97 | N | 54.2 |
|  | 2 | F: GCCAACAACAACAAGGCCAAAC  R: TAGGCTCTGTTGGTGGGAATGT | 118.11 | Low | 393 | N | 58.2 |
| *Candida albicans* | 3 | F: TGCTGAACCGACAGACTCAGTT  R: AAGAGATTGCCAATTGTCCCATA | 187 | High | 104 | CDR2 | 58.5 |
|  | 4 | F: CCTGTTTGAGCGTCGTTTCTC  R: TTTGGTTAGACCTAAGCCATTGTCA | 110.06 | Low | 125 | ITS2;  RDN58 | 56.6 |
| *Chlamydia trachomatis* | 5 | F: CCTGCTGAACCAAGCCTTAT  R: TGATAGCGTCACACCAAGTG | 186 | High | 94 | ompA | 55.1 |
|  | 6 | F: TACGAACTCTTTGTATG  R: TGACTGACTTGTTGGAAA | -15 | Low | 81 | hctA | 41.2 |

**Table S6 Additional experimental details (Instruments, consumables and reagents)**

| **Instruments and consumables** | |
| --- | --- |
| MYFUGE™12 MINI CENTRIFUGE 100-240V (US PLUG) | Benchmark Scientific, Inc |
| GeneRotex 96 Nucleic Acid Extractor | Xi'an Tianlong Science and Technology Co., Ltd |
| Gentier 96E Real-Time PCR System | Xi'an Tianlong Science and Technology Co., Ltd |
| MiniBeadbeater-16 607EUR | BioSpec Products |
| DLAB D1008E | Dalong Xingchuang Experimental Instrument Ltd., China |
| Dry Bath H2O3-PROIII | Coyote Bioscience Yixing Co., Ltd., China |
| Finnpipette F3 1000/200/20/10 | Thermo Fisher Scientific Inc |
| Electrophoresis apparatus DYY-60 | Beijing Liuyi Biotechology Co., Ltd |
| WD-9413B gel imaging system | Beijing Liuyi Biotechology Co., Ltd |
| 0.1 mL 8-strip PCR tubes | Wuxi Guosheng Bio-Eng. Co., Ltd |
| 1.5 mL QSP Snap Cap Microcentrifuge Tubes | Thermo Fisher Scientific Inc |
| 0.1-mm zirconia/silica beads | BioSpec Products |
| **Reagents** | |
| Dnase/Rnase-free Deionized Water (100 mL) | TransGen Biotech Co., Ltd |
| qEx-DNA/RNA | Xi'an Tianlong Science and Technology Co., Ltd |
| SYBR Green (100X) (100 μL/ tube) | Solarbio Co., Beijing, China |
| PCR reaction Solution (100 μL/ tube) | Coyote Bioscience (Beijing) Co., Ltd., Beijing, China |
| DNA maker I MASS (5uL) | TransGen Biotech Co., Ltd |
| 6×DNA Loading Buffer | TransGen Biotech Co., Ltd |

**Methods**

**RNA/DNA extraction and purification**

DNA/RNA was extracted from *C. trachomatis*, *CP*, *Covid-19*, and *HCoV-229* using the Tianlong GeneRotex96 Nucleic Acid Extraction and Purification System with matched EX-RNA/DNA viral nucleic acid extraction kits. 300 µl of fungi (*C. albicans* and *C. tropicalis*) samples and 300 µl of lysate were added to a 1.5 ml screw-capped tube with 0.3 g of 0.1-mm zirconia/silica beads (BioSpec Products). The cell walls were disrupted by centrifugation at 3,450 rpm for 2 minutes. Subsequently both fungal DNAs were extracted and purified using the same instruments and reagents from Tianlong.

**The reaction system and PCR reaction conditions**

PCR reaction was performed in a total volume of 50 μL, including 5 μL DNA/RNA, 0.5 μL SYBR GREEN mastermix (100 X, Solarbio Co., Beijing, China), 0.5 μL primer forward (100 μM) and 0.5 μL primer reverse (100 μM), 32 μL PCR reaction Solution and 11.5 μL Dnase/Rnase-free deionized water. The reaction was carried out using the Gentier 96E Real-Time PCR System. The program included pre-denaturation at 42°C for 5 minutes, followed by a two-step PCR amplification process (Stage 1: 95°C for 1 minute; Stage 2: 95°C for 10 s, 60°C for 30 s, for 45 cycles), and concluded with a melt curve stage at 95°C for 1 minute, 60°C for 15 s, and 98°C for 5 s.

**Agarose Gel Electrophoresis**

DNA integrity and quality were evaluated by agarose gel electrophoresis (concentration of agarose gel: 2%; voltage: 180 V; electrophoresis time: 70 min) with DYY-60 electrophoresis apparatus (Beijing Liuyi Instrument Factory, Beijing, China). The images were captured by WD-9413B gel imaging system (Beijing Liuyi Instrument Factory, Beijing, China) at 302 nm.

**Result**

1. **Amplification curve**

The results of the amplification curve analysis are illustrated in Figure S1. All high-specificity primers successfully amplified the target species, as evidenced by the red curves in Figures S3A, S3B, and S3C. It is noteworthy that no nonspecific amplification was detected for the specified species, including *HCoV-229*, *C. tropicalis*, and *CP*, as indicated by the blue curves in Figure S3A, S3B, and S3C, respectively. However, some of the low-specificity primers displayed non-specific amplification, as illustrated by the blue curves in Figures S3D and S3E. Regrettably, no PCR amplification was achieved when using a low-specificity primer for *C. trachomatis* (Figures S3F). The details of cycle threshold (CT) values from specific and nonspecific amplification are shown in Table S7.

**
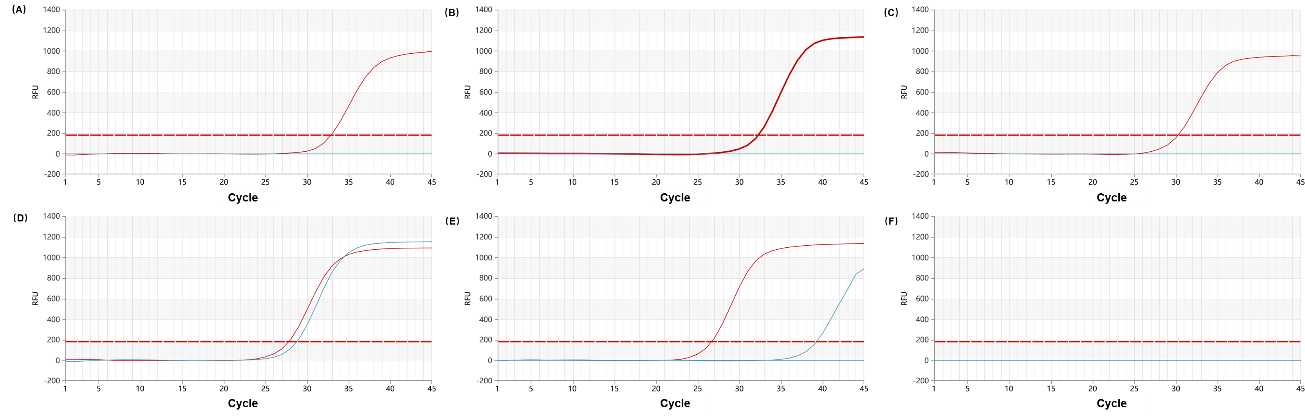
**

**Figure S****3 Amplification curve results.** (A) Amplification of *Covid-19* (red) and *HCoV-229* (blue) using *Covid-19* high-specific primer pairs. (B) Amplification of *C. albicans* (red) and *C. tropicalis* (blue) using *C. albicans* high-specific primer pairs. (C) Amplification of *C. trachomatis* (red) *and CP* (blue) using *C. trachomatis* high-specific primer pairs. (D) Amplification of *Covid-19* (red) and *HCoV-229* (blue) using Covid-19 low-specific primer pairs. (E) Amplification of *C. albicans* (red) and *C. tropicalis* (blue) using C. albicans low-specific primer pairs. (F) Amplification of *C. trachomatis* (red) and *CP* (blue) using C. trachomatis low-specific primer pairs.

**Table S7 Cycle threshold (CT) values from specific and non-specific amplification**

| **Species** | **Primer No.** | **Specificity** | **Cycle threshold (Ct) value** | | |
| --- | --- | --- | --- | --- | --- |
|  |  |  | ***Covid-19*** | ***HCoV-229*** | **NTC** |
| *Covid -19* | 1 | High | 32.902 | - | - |
|  | 2 | Low | 27.848 | 28.816 | 27.723 |
| **Species** | **Primer No.** | **Specificity** | ***C. albicans*** | ***C. tropicalis*** | **NTC** |
| *Candida albicans* | 3 | High | 32.332 | - | **-** |
|  | 4 | Low | 26.715 | 39.270 | **-** |
| **Species** | **Primer No.** | **Specificity** | ***C. trachomatis*** | ***CP*** | **9NTC** |
| *Chlamydia trachomatis* | 5 | High | 30.285 | - | - |
|  | 6 | Low | - | - | - |

1. **Melting curve**

The melting profiles were displayed as normalized melting peaks (Figure S2). High-specific primers were utilized for *Covid-19*, *C. albicans*, and *C. trachomatis* to generate specific amplification products for these organisms. The melting curve profiles displayed single peaks, indicating the absence of primer dimerization or non-specific amplification (illustrated by the red curves in Figure S4A, S4B, and S4C). Meanwhile, the non-specific amplification of *HCoV-229*, *C. tropicalis*, and *CP* showed no amplification products, as shown by the green curves in Figs. S4A, S4B, and S4C, validating the high specificity of these primer pairs. The melting curves for the low specificity primers show different results. In Figure S4D, for the *Covid-19* specific amplification, the melting curve shows three peaks highlighted by the red curve. Conversely, non-specific amplification of *HCoV-229* and the no template control (NTC) show an abnormal single peak, represented by the blue and green curves, respectively. In the case of the low-specific primer for *C. albicans*, a single peak was observed during *C. albicans* amplification, and a smaller single peak was detected during nonspecific amplification of *C. tropicalis* (see Figure S4E). However, for the low-specificity primers of *C. trachomatis*, no amplification products were obtained for *C. trachomatis* (specific amplification) or *CP* (nonspecific amplification), as illustrated in Figure S4F.

**
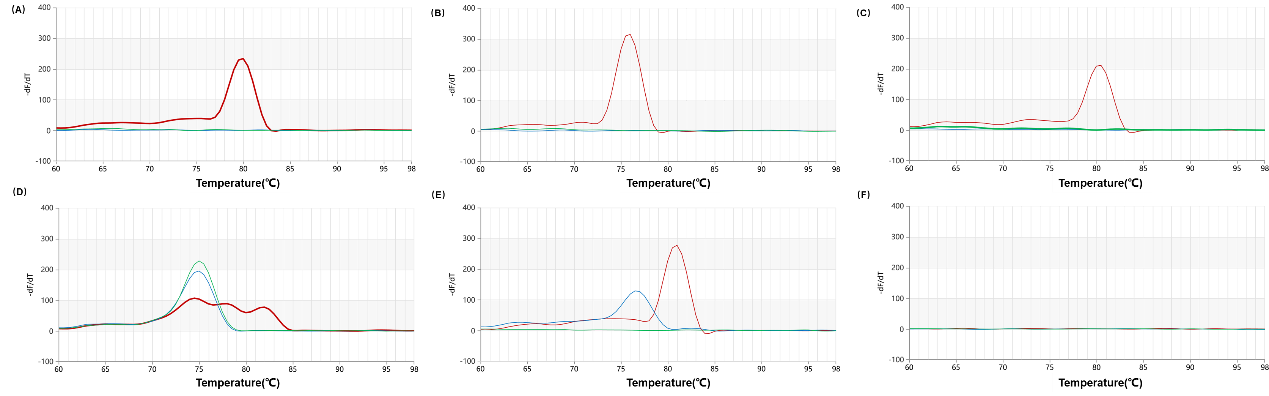
**

**Figure S4 Melting curves PCR results.** The red curve represents the PCR product of specific amplification, whereas the blue curve depicts the PCR product of non-specific amplification, and the green curve indicates the PCR product obtained from the no template control (NTC). Each subfigure represents the melting curves with different primer pairs. (A) *Covid-19* high-specific primer. (B) *C. albicans* high-specific primer. (C) *C. trachomatis* high-specific primers. (D) *Covid-19* low-specific primer. (E) *C. albicans* low-specific primer. (F) *C. trachomatis* low-specific primers.

1. **Agarose gel electrophoresis**

Agarose gel electrophoresis results from RT-qPCR experiments align with the findings of the melting curve analysis (see Figure S5). The species-specific primers successfully amplified the intended target species, resulting in PCR amplicon sizes of 97 bp for *Covid-19*, 104 bp for *C. albicans*, and 94 bp for *C. trachomatis*. However, the lack of specificity in the primers for *Covid-19* and *C. albicans* led to unintended amplification of PCR products under 100 bp potentially including primer dimers. Notably, no electrophoretic bands were observed in either the target or non-target species when using the low specific primers of *C. trachomatis*.

**
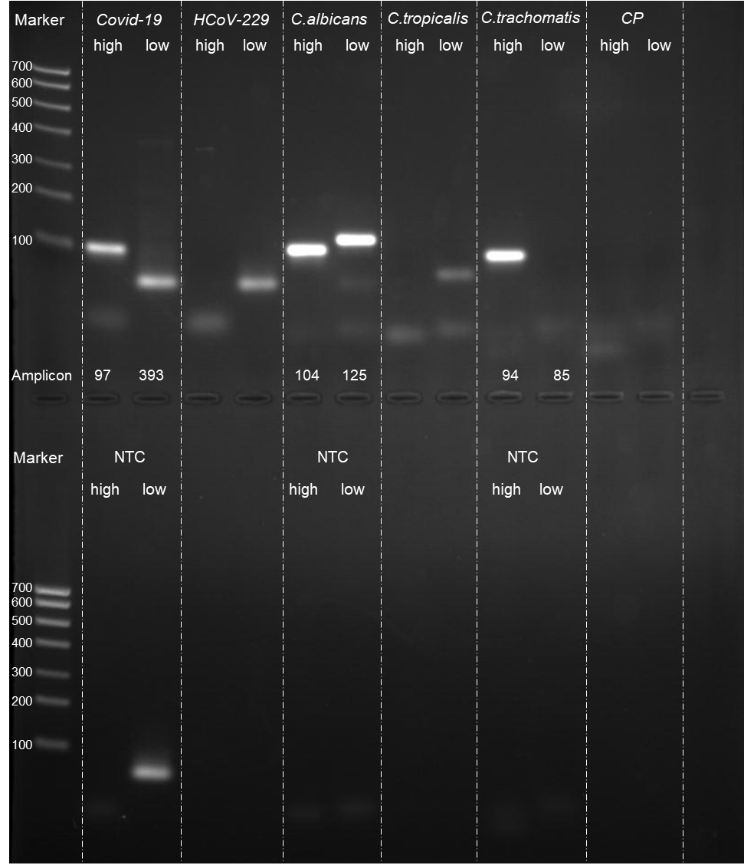
**

**Figure S5 Agarose gel electrophoresis results.**
