# Supplementary figures and images for: MiPRIME: an integrated and intelligent platform for mining primer and probe sequences of microbial species
Source: Bioinformatics. 2024 Jul 2;40(7):btae429. doi: 10.1093/bioinformatics/btae429 (PMC11246166; doi:10.1093/bioinformatics/btae429)

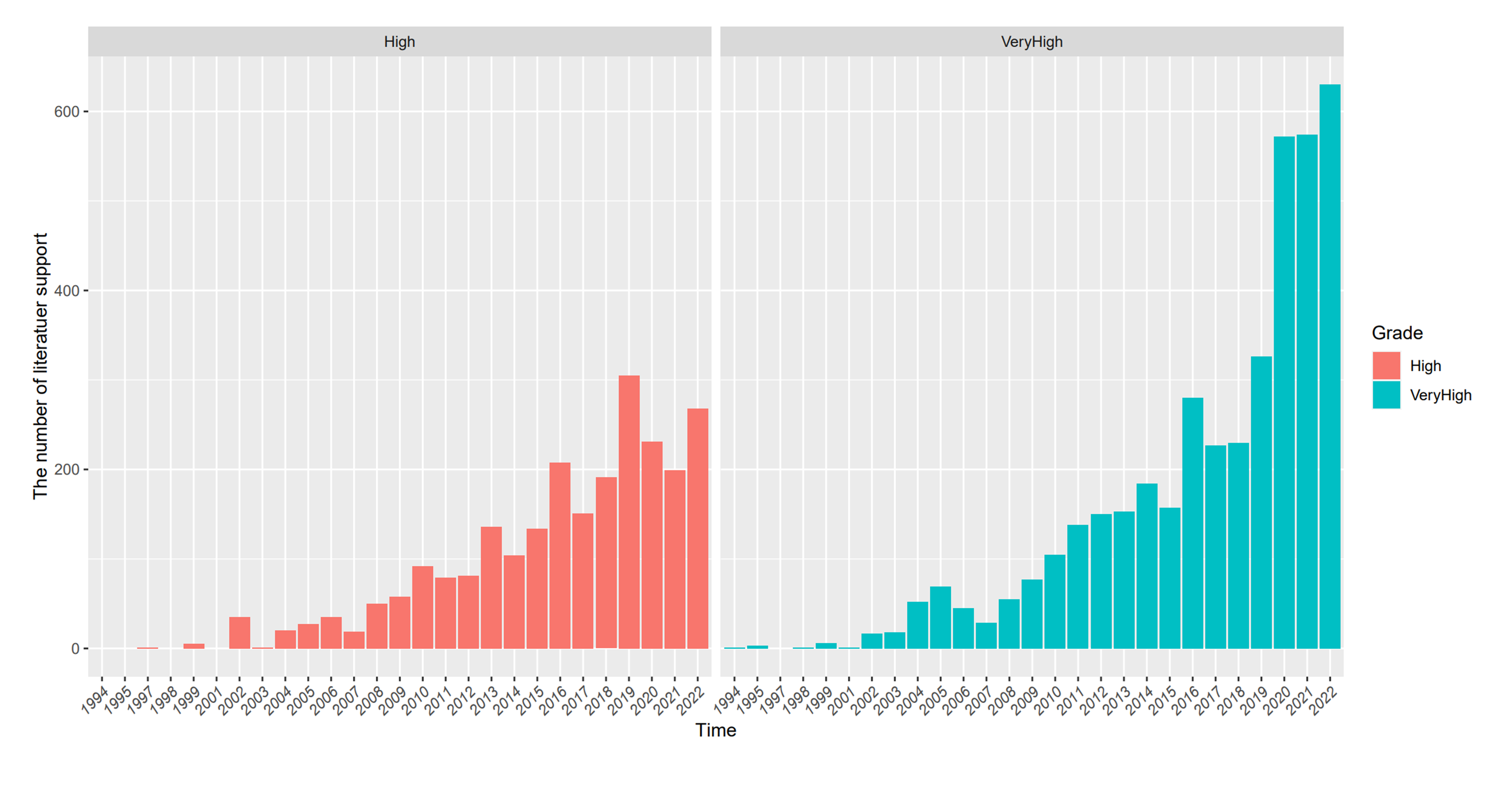

Supplement: btae429_Supplementary_Data [file btae429_supplementary_data.zip › SI-FIGURE1.png]
